# Ubiquitous Amyloids

**DOI:** 10.1007/s12010-012-9549-3

**Published:** 2012-02-19

**Authors:** Wojciech Pulawski, Umesh Ghoshdastider, Vincenza Andrisano, Slawomir Filipek

**Affiliations:** 1 Faculty of Chemistry, University of Warsaw, ul. Pasteura 1, 02-093 Warsaw, Poland; 2 Centre for Biomolecular Magnetic Resonance, Institute for Biophysical Chemistry, Goethe-University of Frankfurt/Main, Frankfurt/Main, 60438 Germany; 3 International Institute of Molecular and Cell Biology, ul. Ks. Trojdena 4, 02-109 Warsaw, Poland; 4 Department of Pharmaceutical Sciences, University of Bologna, via Belmeloro 6, 40126 Bologna, Italy

**Keywords:** Amyloids, Prions, Fibrils, Amylome, Amyloidogenic segments, Aging diseases

## Abstract

The common view of amyloids and prion proteins is that they are associated with many currently incurable diseases and present a great danger to an organism. This danger comes from the fact that not only prion proteins, but also the infectious form(s) of amyloids, as it has been shown recently, are able to transmit the disease. On the other hand, organisms take advantage of the strength and durability of specific forms of amyloids. Such forms do not spread any disease. Also, in nanotechnology there is a constantly growing need to employ amyloid fibrils in many industrial applications. With increasing knowledge about amyloids and prion proteins we are aware that the amyloidal state is inherent to any protein, making the problem of amyloid formation a central one in aging-related diseases. However, the “good” amyloids can be beneficial and even necessary for our health. Furthermore, because of their mechanical properties, the amyloids are of great interest to engineers.

## The World of Amyloids—Amylome

Many proteins can convert into amyloid fibrils either to comply with the physiological needs or as part of a pathological scenario. To fight against pathological amyloid states and to stop growth of particular amyloids, the prospective inhibitors of amyloid fibril formation may be helpful. Unfortunately, the structure-based drug design is hampered because amyloid proteins do not have defined structures. Nonetheless, in a recent paper [1], the Eisenberg and Baker groups described a structure-based design of such inhibitors. They demonstrated [1] that a structure of a short segment directly engaged in fibril formation can be sufficient for the design of fibril formation inhibitors and that the computational methods may be successful in designing novel peptide–peptide interfaces. The inhibitory peptides were designed employing modeled structures of the so-called “steric zippers” which are dual β-sheets. One of the inhibitory peptides, consisting exclusively of d-amino acids, inhibited the formation of the tau protein tangles associated with Alzheimer’s disease [2]. Its target was a hexapeptide VQIVYK corresponding to tau protein residues 306–311. This fragment was shown to be important for fibril formation by the full-length tau protein [3, 4], and fibrils formed by this fragment are similar to full-length tau fibrils. The researchers also designed a non-natural l-amino acid inhibitor of the amyloid fibril enhancing transmission of HIV. Its target was also a steric zipper structure of the GGVLVN peptide from a fragment of prostatic acid phosphatase [5]. The authors designed the specific and tight interface between the inhibiting peptide and the end of the steric zipper by maximizing the number of hydrogen bonds and hydrophobic interactions.

Eisenberg also introduced the concept of amylome [6] defined as a large set of proteins capable of forming amyloid-like fibrils. It was suggested in this paper that the amyloid state is accessible to many more proteins that was originally thought—not only to those whose entire sequence is engaged in amyloid formation. In the classical view, in each disease of amyloid origin, one or two fibril-forming proteins were characterized, namely β-amyloid and tau proteins in Alzheimer’s disease, α-synuclein in Parkinson’s disease, huntingtin polyglutamine stretch in Huntington’s disease, prion protein in Creutzfeldt-Jakob disease and amylin in type II diabetes [7]. Aggregates of these proteins are toxic, highly stable, and are producing polymer-like amyloids by recruiting normal, soluble proteins [8].

Eisenberg and coworkers [6] investigated the factors that enable a protein to acquire an amyloidal form. It turned out that the major factor responsible for amyloid formation is the presence of a segment in the protein that can form a tightly complementary interface with other mostly identical segments. Such interface between the segments was named “steric zipper.” It is usually created by self-complementary β-sheets that form the amyloid fibril. Another suggested factor is a sufficient conformational freedom of the self-complementary segment allowing for interaction with other identical segments. Eisenberg’s group examined more than 12,000 proteins whose folded, three-dimensional (3D) structures are already known. The predictions of an amyloid state were done by the modified 3D-Profile method [9] based on the crystal structure of the NNQQNY motif, known to form a steric zipper. They computationally examined proteins of three organisms: *Escherichia coli, Saccharomyces cerevisiae*, and *Homo sapiens*. The method identified protein segments with high tendency to form amyloid fibrils and demonstrated that a specific residue order is required for fiber formation. These segments were typically about six amino acids long and could be exposed for instance during thermal motion of the protein. It was found that 95% of the predicted amyloid-prone segments are buried within the protein, and those that are exposed are too twisted and inflexible to form a “steric zipper” with partner segments. Using bovine pancreatic ribonuclease A (RNase A) as a model system, they experimentally validated the accuracy of predictions and investigated the effect of sequence and residue composition. For instance, the FERQHM sequence was one of several segments predicted and experimentally confirmed not to form fibrils. However, when the residues of this segment were rearranged to QEMRHF, the energy of the rearranged segment fell below the formerly estimated threshold of −23 kcal/mol; QEMRHF was thus predicted to form fibrils, which was subsequently confirmed by EM images. On the contrary, the fibril-forming segments QANKHI and STMSIT were rearranged to IHKAQN and ISMTTS, respectively. The rearranged sequences were predicted not to form fibrils, and it was also confirmed by experimental methods. Such shuffling experiments suggest that the tendency to form amyloid-like fibrils is strongly sequence-dependent and relatively insensitive to amino acid composition.

In earlier research it has also been shown [10, 11] that many globular proteins can be converted to the amyloid state by a variety of denaturing processes, suggesting that conversion may generally be applicable to all proteins. The self-association of peptides and proteins into well-ordered supramolecular structures is of central importance in normal physiological processes such as the assembly of collagen fibrils [12, 13], actin filaments [14] but also in pathophysiological cases [15]. Integration of old and new techniques and development of novel methods of nanoscience can provide powerful opportunities to increase our understanding of processes underlying amyloid-related disorders [16]. Until recently, it was commonly believed that amyloid formation is a feature of only a tiny fraction of proteins. Not all proteins, however, form amyloids because in most cases these potentially harmful segments are hidden deep inside the protein structure and are kept under control. Such behavior can be of evolutionary origin suggesting that evolution treats amyloids as a fundamental threat. The presence of different kinds of amyloids have been confirmed in some of the most common age-related diseases, so one can suppose that the accumulation of amyloid is unavoidable during aging. Sometimes, the presence of amyloid deposits does not give rise to neurodegenerative symptoms, indicating that amyloid fibrils do not cause the onset of disease. Therefore, one of the hypotheses suggested that the oligomeric intermediates are the toxic species while the fibrils are detoxification products [17]. Fibrils are not the only shape taken by amyloids especially during the nucleation process. For instance, spheroidal oligomeric species have been demonstrated for α-synuclein—they are thought to be responsible for cytotoxicity towards the neuronal cells observed in Parkinson’s disease [18, 19].

On the basis of current research, it was proposed by Eisenberg [6] that the amyloid state is more like a default state of a protein especially in the absence of specific protective mechanisms such as chaperoning. Proteins that are not correctly folded and less protected (by chaperoning and/or disposal mechanisms) are predisposed to become amyloids. The amyloid-associated diseases that are known so far probably involve only the most vulnerable human proteins. Many research groups try to find ways to supplement or boost the protective mechanisms, in the hope of treating or preventing the original cause of amyloid-linked diseases. Even a subtle pharmacological interference in the process of amyloidogenesis might have a major effect on the disease and even on ageing in general. On the other hand, one can enhance the natural protective mechanisms that stabilize a protein. A review of potential strategies for tackling protein aggregation and the toxicity associated with it has been published by Bartolini and Andrisano [20]. However, the complexity of the aggregation processes and other related events account for the fact that no effective treatments for these disorders are currently available. Studies of the structures of amyloids and mechanisms of amyloid formation should unveil new molecular targets for potential anti-neurodegenerative drugs. Although the three characteristic stages of nucleation-dependent fibrillation—seed formation, accelerated fibrillar growth, and the stationary phase—have been examined separately, additional studies are required to unambiguously uncover the mechanism of amyloidogenesis.

## Molecular Structures of Amyloids

Amyloid fibrils represent an energetically stable state of many proteins and peptides. Basically, amyloid fibers are a bundle of highly ordered filaments composed of ladders of β-strands that are placed perpendicular to the fiber axis and are arranged in hydrogen-bonded β-sheets [21]. Amyloid fibers have a diameter of about 7–10 nm and can be up to several micrometers long. In cross sections, amyloid assemblies appear as hollow cylinders or ribbons. The measurements of amyloid fibers revealed that their strength is comparable to that of steel while their mechanical stiffness matches that of silk [22]. In general, amyloid structures attain their stability through non-covalent bonds, mainly hydrogen bonds stabilizing the β-sheets, but also through hydrophobic and π–π stacking interactions of the side chains. The frequent occurrence of aromatic residues in short amyloid-related peptides suggests that π stacking may play a role in speeding-up the self-assembly process by providing geometrical constraints that promote directionality and orientation of the growing fibril. The importance of hydrogen bonds is especially seen in glutamine- and asparagine-rich proteins which form amyloids. Extended sequences of repeated glutamine (or asparagine) units are related to several amyloidoses such as Huntington’s disease and spinocerebellar ataxia, and also to the aggregation of yeast proteins into prions.

**Fig. 1:**
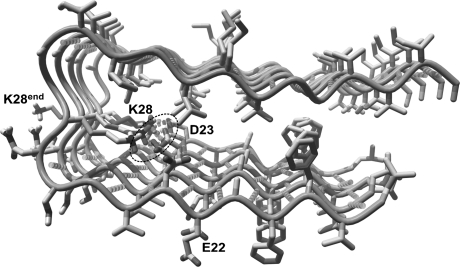
The structure of fragment of β-amyloid (Aβ_42_) obtained by NMR methods (PDB code 2BEG). The salt bridge K28-D23 is linking adjacent β-sheets, therefore, the residues K28 and D23 from terminal strands are unpaired (marked). A salt bridge K28-D23 was marked with a *dotted ellipse*. Hydrogen bonds shown as *dashed cylinders*

The three-dimensional structure of the fibrils comprising Aβ_42_ (Protein Data Bank code 2BEG) was obtained using quenched hydrogen/deuterium exchange NMR in solution, while the β-sheet arrangement was taken from previous solid-state NMR studies of this structure. Residues 18–42 form a β–strand–turn–β–strand motif while residues 1–17 are disordered and could not be detected. The parallel β-sheets are formed by residues 18–26 (β_1_ strand) and 31–42 (β_2_ strand). The repeating structure of a protofilament requires two monomers because of the salt bridge D23-K28 formed between adjacent monomers. This interaction pattern leads to the formation of partially unpaired β-strands at the ends of the Aβ_42_ fibrils (Fig. 1). Such unpaired ends explain the specific shape of these fibrils and could be a target for inhibitors of fibril growth [23]. The salt bridge and also the hydrophobic interactions of the side chains keep the structure rigid and compact despite the repulsion between the charged residues E22, D23, and K28 from the adjacent β-strands.

**Fig. 2:**
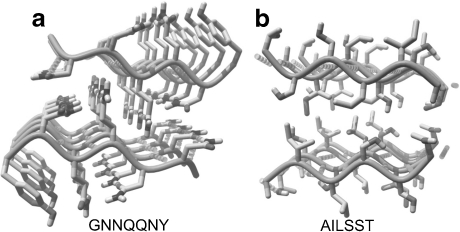
The crystal structures of amyloids **a** GNNQQNY (PDB code 2OMM), adjacent β-strands are parallel, and **b** AILSST (PDB code 3FOD), adjacent β-strands, are antiparallel. Hydrogen bonds shown as *dashed cylinders*

Amyloid fibrils can be formed by different proteins and usually contain a common cross-β spine. Glutamine repeats were first suggested to act as “polar zippers” joining monomeric units together and propagating the amyloid fibrils. The structure of the fibril-forming segment, GNNQQNY, of the yeast prion protein Sup35 has been recently revealed by crystallography [24]. It is formed by a pair of β-sheets, with the facing side chains of the two sheets locked together in an interdigitated way forming a so-called “dry steric zipper” (Fig. 2a). Eisenberg and coworkers [24] reported dozens of other segments from fibril-forming proteins that are able to form amyloid-like fibrils on their own. The segments from the β-amyloid and tau proteins, the PrP prion protein, insulin, islet amyloid polypeptide (IAPP), lysozyme, myoglobin, α-synuclein, and β-2-microglobulin were analyzed. The obtained structures are characterized by structural features that are shared, at the molecular level, by all the proteins studied but some variations in the atomic architecture of the amyloid-like fibrils can provide some clues on their origin and the mode of growth. In the GNNQQNY amyloid, the peptide strands are parallel, and the Asn and Gln residues form regular rows connected by hydrogen bonds in addition to the hydrogen bonds in the β-sheet. The hydrophilic character of these residues and their length make the steric zipper interface highly interdigitated. In the other amyloid formed from the AILSST peptide (Fig. 2b), the strands are antiparallel, and the steric zipper interface is formed mostly by hydrophobic residues Ile and Leu. The hydrogen bonds between side chains of serine residues are bridged by water molecules.

According to [24] there are eight types of the steric zipper interfaces classified according not only to the orientations of their strands (parallel or antiparallel) but also faces (face-to-face or face-to-back arrangement) and the up or down orientations of the edges of the strands. So identical peptides can form different polymorphic structures characterized by distinctive phenotypes. New polymorphic crystal structures of segments of the prion and other amyloid proteins [25] proved to be useful for elucidating the structural mechanisms of different modes of fibrillation. Additionally, β-sheets formed by the same segment of a protein can reveal alternative packing arrangements (polymorphs). Such polymorphism can be responsible for enduring conformations capable of “encoding” prion strains. Such transfer of protein-encoded information into prion strains involves sequence specificity and recognition by means of noncovalent bonds.

## Mechanisms of Amyloid Formation

Amyloid fibril formation is considered to be a signature of neurodegenerative processes. The exact processes leading to cellular degeneration remain unknown although several amyloid-involving mechanisms have been proposed [26]: (1) amyloids occupy the extracellular space and destroy the structure of cells and tissues, (2) amyloid fibrils destabilize cell membranes, (3) heavy metals incorporate into amyloids and generate reactive oxygen compounds which affect cellular functions, (4) some proteins essential for cell survival are trapped in protein aggregates. In a recent review, Zerovnik et al. [27] classified the mechanisms by which proteins undergo ordered aggregation into amyloid fibrils: (1) templating and nucleation; (2) linear, colloid-like assembly of spherical oligomers, and (3) domain swapping. The local environment and inter- and/or intra-molecular interactions may have a significant influence on the conformation of certain amino acid residues. Therefore, even small variations in pH, temperature, and ionic strength could induce changes in the conformational propensities of these residues (leading to a different secondary structure) including their ability to aggregate.

**Fig. 3:**
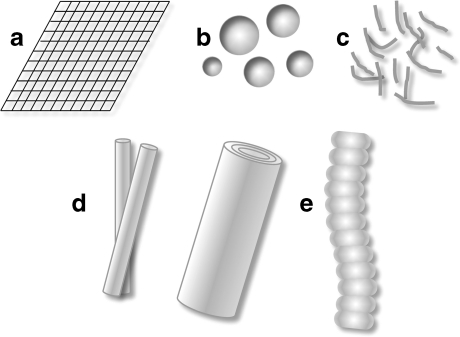
Different forms of amyloids: **a** squared plates, **b** nanospheres, **c** hydrogels, **d** tubular structures—single-walled and multi-walled tubes, **e** fibrils

Some proteins forming amyloids, for instance α-synuclein which contributes to the formation of intracellular Lewy bodies in Parkison’s disease [28], can exist without a defined structure. It was postulated that exogenous α-synuclein fibrils induce the formation of Lewy body-like intracellular inclusions [29]. Other proteins with an unordered structure are the IAPP in type II diabetes [30] and β-amyloid in Alzheimer’s disease [31]. Such an unfolded structure allows the protein to be rather easily self-assembled into fibrils. On the other hand, some amyloidogenic proteins preserve their 3D structure until the actual fibrillation [11]. This group of proteins includes β-2 microglobulin identified in dialysis-related amyloidosis [32], huntingtin in Huntington’s disease [33], immunoglobulin V_L_ domain in light-chain amyloidosis [34], lysozyme in hereditary systemic amyloidosis [35], prion protein in Creutzfeldt-Jakob disease [36], and transthyretin in senile systemic amyloidosis [37]. However, regardless of the initial structure, the amyloid fibrils obtained from different amyloidogenic proteins and peptides are very similar and adopt a cross β-sheet conformation [10] even though these proteins and peptides share rather little amino acid sequence similarity. It was known that even all α (protein composed of α-helices only) or mixed α/β protein types can form β-sheet fibrils. Therefore, it was tempting to suggest that when elucidated for a given protein in a particular disease, the molecular mechanism of amyloidogenesis will apply to other proteins and amyloid-related diseases. However, it became gradually recognized that amyloid fibrils exist in multiple fibrillar forms and exhibit so-called fibrillar polymorphisms. Even a single amyloidogenic protein can create multiple forms of amyloid fibrils depending on the conditions in which fibrillation occurred. This may indicate that amyloidogenesis can proceed via multiple mechanisms. Various types of possible amyloids structures are shown on Fig. 3.

The conversion and aggregation of proteins from their soluble states into well-organized fibrils is associated with a wide range of conditions, usually pathological, including neurodegenerative diseases and amyloidoses. Although a conformational change of the protein native state is generally necessary to initiate aggregation, it was shown that a transition across the large unfolding energy barrier is not essential and that the aggregation may be initiated from locally unfolded states that become accessible, for example, via thermal fluctuations occurring under physiological conditions [11]. Conformational states thermodynamically distinct from the native state, but structurally similar to it, can be easily accessed from the native state through thermal fluctuations. These states are separated from the native state by a relatively low energy barrier. They are therefore only transiently populated under physiological conditions, yet they can be sampled more frequently than the entirely unfolded state (global unfolding) or a partially folded state. The existence of such conformational states can be deducted from the observation that, under physiological conditions, the amide hydrogen atoms buried in the interior of a native protein can exchange with the solvent hydrogen atoms more rapidly than it could be expected from the rate of protein unfolding. The possibility of sampling of such partially unfolded states is also confirmed by long molecular dynamics simulations.

**Fig. 4:**
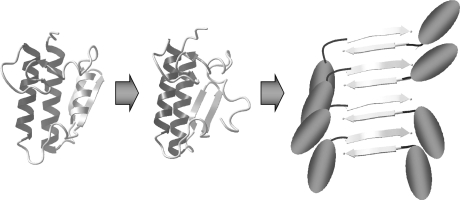
A scheme displaying the process of amyloid formation resulting from instability of part of the protein structure (*light gray helix*). The rest of the proteins not participating in amyloidal β-sheet development are shown as *ellipses*. All structures of proteins were created in Yasara (YASARA Biosciences)

Amyloid self-polymerization is also the basis of the “protein-only” hypothesis for the mechanism of prion infectivity. The infectious prion conformation replicates itself in a host by pairing with the host protein and forcing it into the infectious, fibrillar conformation. It was found that amyloids, including β-amyloid, can also be infectious like the PrP^Sc^ prion protein. Data showed that β-amyloid, which is associated with Alzheimer’s disease, behaved like an infectious agent when injected into the brain of a mouse. The same mechanism was suggested in the case of other diseases in which amyloid forms of proteins were detected [38]. A self-complementary “steric zipper” structure identified in protein fibrils allows them to tangle very tightly with an identical segment exposed on another protein. Several of these segments are needed to seed, or nucleate, an amyloid. Segments attach to one another and form fibrils. As they grow, fibrils are fringed by the remnants of the host protein segments (Fig. 4). Eventually, this developing fibril breaks to form two smaller fibrils, each of which starts to grow at both ends again. The nucleation events are rare but once the fibril is formed its spreading is fast. Ohhashi et al. [39], based on mutational and biophysical analyses, proposed that before fiber formation, the prion domain (Sup35NM, consisting of residues 1-254) of yeast prion Sup35 forms oligomers in a temperature-dependent reversible manner. Experiments revealed that “non-native” aromatic interactions outside the amyloid core drive oligomer formation by bringing together different monomers, which leads to the formation of new amyloid cores. In this way, the transient non-native interactions in the initial nucleus are responsible for the diversity of amyloid conformations.

## Kinetics of the Growth of Amyloid Fibrils

Using quantitative measurements of protein aggregation rates, Buell et al. [40] developed a kinetic model of a conversion of a protein from a soluble to a fibrillar form which shows that there is a single free energy aggregation barrier controlling the addition of protein molecules into amyloid fibrils. Other characteristics of the aggregation process are natural consequences of finite diffusion times. These findings suggest that this process does not follow a simple chemical mechanism, but rather operates in a way analogous to the multitrajectory (landscape) models of protein folding defined by stochastic dynamics on the surface of the potential energy of the system. Another kinetic study [41] was based on quantitative quartz crystal microbalance measurements of the kinetics of the growth of amyloid fibrils in crowded environments. Such environments strongly modify the association of components, through attractive entropic interactions such as the depletion pressure that results from the entropically favorable overlap of the regions surrounding two aggregating particles. The complex effects of macromolecular crowding on the growth of amyloid fibrils can be described on the basis of established physical principles using a combination of osmotic effects and entropic interactions. Within this framework, it was possible to predict the aggregation susceptibility of many proteins with different structural properties. Campioni et al. [42] described two types of oligomers formed by the HypF-N protein (91-residue N-terminal domain of *E. coli* HypF) that are morphologically similar, as detected with atomic force microscopy and thioflavin T assays, though one is benign when added to cell cultures, whereas the other is toxic. They found that a lower degree of hydrophobic packing is correlated with a higher ability to penetrate the cell membrane and to cause an influx of calcium ions. It suggests that structural flexibility and hydrophobic exposure are primary determinants of the ability of oligomeric assemblies to cause cellular dysfunction and its consequences such as neurodegeneration. A broad review on aggregation kinetics and mechanisms of fibril formation was prepared by Morris et al. [43]. By employing an extensive mathematical framework, the authors revealed different aspects of nucleation, growth, and disintegration of various amyloid intermediates.

## Specific Mechanisms of Fibrillation

To explain the process of α-synuclein amyloidogenesis, a specific mechanism named double-concerted fibrillation, corresponding to the prevailing nucleation-dependent fibrillation model, was introduced [44]. According to the double-concerted fibrillation, the amyloid fibril formation is achieved via two consecutive, concerted associations of monomers and the subsequently formed oligomeric granules. These newly formed oligomeric species act as units for fibril formation and subsequent growth in the absence of a template [26]. Template-dependent fibrillation requires a pre-existing fibril to which the incoming protein monomers or granules can attach if, due to a conformational change, they match the structure of the template. The fibril is extending, and the subsequent assembling step requires the exposure of the interactive domains of the protein to facilitate further molecular self-assembly. Template-dependent fibrillation is the most appropriate mechanism to study the infectivity of prion proteins. Prion protein (PrP^C^) is anchored to the cellular surface via the glycosylphosphatidylinositol moiety. Its conformational change into another structural entity (PrP^Sc^) is associated with the occurrence of transmissible spongiform encephalopathies (a group of prion diseases). Exogenous PrP^Sc^ directs the conversion of PrP^C^ into PrP^Sc^ conformation by acting as a template. In a template-independent fibrillation, the amyloidogenic conformations of building units are induced (by physical or chemical influences) before the main molecular assembly occurs. Polymorphism of amyloids, reflected by the existence of various types of amyloid fibrils, especially in the presence of specific ligands, is achieved via multiple pathways. The natively or partially unfolded amyloidogenic proteins are at a high-energy state, but increased conformational entropy could allow the self-interactive conformers to be stabilized. Initial stable seed formation is by no means privileged since the production of an oligomeric nucleus is an entropically expensive process which needs to be overcome by an enthalpic advantage [26].

Another mechanism, the 3D domain swapping, has been suggested to explain the development of protein oligomer assembly of cystatins and stefins [27]. These small globular proteins (11–13 kDa) are part of a large family of cysteine proteinase inhibitors which are also linked to amyloid diseases. The process of domain swapping is rate limiting for the initiation of amyloid fibril formation because of a high energetic barrier in this process. Nevertheless, it was suggested that, in principle, any protein is capable of oligomerization by 3D domain swapping [45]. Guo and Eisenberg [46] proposed the term “run-away domain-swapping” for a process of continuous domain swapping. Wahlbom et al. [47] used the term “propagated domain-swapping” to describe a similar process of continuous domain swapping in the formation of cystatin C prefibrillar oligomers and fibrils.

Apart from the oligomeric species formed on the route to mature fibrils the off-pathway oligomers are also formed. They are the dead ends of an alternative folding pathway because they are incapable of converting directly to fibrils and substantially slow fibril formation. The OFF model for amyloid formation was first described by Pallitto and Murphy [48]. In this model, denatured monomers are refolded into either stable monomers or dimers or less stable dimeric intermediates which can form non-fibrillar oligomeric forms. These initial steps are followed by a cooperative assembly of the fibril-prone dimeric intermediates into a nucleus from which the protofibrils originate.

## Conformationally Distinct Amyloid States

Since it was known that the amino acid position specifically contributes to protein oligomerization, Maji et al. [49] performed amino acid substitution to determine the distribution frequency of the Aβ oligomer. The substitutions were done at positions 1, 10, 20, 30, and 40 (for Aβ_40_) or 42 (for Aβ_42_). The effects of these mutations were probed using circular dichroism spectroscopy, thioflavin T binding, electron microscopy, and other techniques. All peptides displayed a transition from a random coil to α/β and to all-β structure, but substitution-dependent changes in the kinetics of assembly and the complexity of conformers were observed. The ability of a single substitution (Tyr in position 1) to alter the Aβ assembly kinetics and the oligomer frequency distribution suggests that the N-terminus is also involved in the oligomerization process and that, most probably, there is a competition between the N- and C-termini to form a stable complex with the central hydrophobic cluster. Additionally, recent electron microscopy and AFM data for Aβ_40_ suggest that dimerization and subsequent monomer attachment are processes in which significant conformational changes occur in the monomer. It was also found that dimers were threefold more toxic than monomers, and tetramers were about 13-fold more toxic [50].

Using mass spectrometry and ion mobility spectrometry Bowers and coworkers [51] investigated a mixture of Aβ_40_ and Aβ_42_. A heterooligomer was formed composed of equal parts of both forms of Aβ. These mixed species comprise an oligomer distribution extending to tetramers, similar to the structures created by Aβ_40_, whereas Aβ_42_ alone produced longer oligomers (dodecamers) indicating that Aβ_40_ inhibits oligomerization of Aβ_42_. In solution, Aβ_40_ and Aβ_42_ adopted similar random coil structures; however, Aβ_42_ was significantly more neurotoxic and formed amyloid fibrils much more rapidly than the shorter form of Aβ. Although amyloid formation is triggered by a transient nucleus, the mechanism by which the initial nucleus is formed and allows the protein to acquire a specific amyloid conformation is still unclear. The observation that Aβ_40_ and Aβ_42_ self-assemble via different pathways put forward the Aβ_42_ dodecamers as candidate primary toxic species in Alzheimer’s disease [52].

If mutations in sequence or changes in environmental conditions elicit partial unfolding of the native state of a protein, the protein will tend to aggregate, sometimes into fibrillar structures. The metastable, partially unfolded states that precede the aggregated states of proteins are of special interest because of their specific features and especially because of increased toxicity. It was found that protein aggregation is favored by conditions that promote stable intermolecular interactions, particularly the hydrogen bond formation. Calamai et al. [53] showed that human muscle acylphosphatase is able to form both fibrillar and non-fibrillar aggregates with a high β-sheet content from partially unfolded states with very different structural features due to the use of different destabilization factors: urea or increased temperature followed by incubation in the presence of different concentrations of 2,2,2-trifluoroethanol (solvent that has been found to promote aggregation of other polypeptides, including the natively unfolded Aβ peptide). The same amino acid sequence can give rise to several conformationally distinct amyloid states. To address this puzzle, Ostapchenko et al. [54] studied two amyloid states of the prion protein (referred to as R- and S-fibrils). The obtained results suggested that the energy landscape for protein folding and assembly contains several close to global free-energy minima: one of which is occupied by the native state and the remaining ones by the amyloid states. The transmissible form of prion disease can be induced in wild-type animals by inoculation with R-fibrils while S-fibrils failed to induce the prion disease. Recently, an apparent generation of toxic prions (PrP^Sc^) in normal brain tissue in the presence of metal (steel wires) has been discovered. The metal catalyzed de novo formation of PrP^Sc^ from a normal cellular prion protein [55]. Alternatively, metal surfaces might concentrate the already existing PrP^Sc^ to the extent that it became quantifiable by the cell assay.

## Molecular Simulations of Amyloids

The long time scale in which the aggregation takes place is prohibitive for molecular dynamics (MD) simulations. However, some structural and dynamic features of amyloids were investigated using coarse grain protein models and specific MD or Monte Carlo procedures. Urbanc et al. [56] elucidated the structural characteristics of oligomers of Aβ_40_ and Aβ_42_ and of their mutants. They simulated oligomer formation using discrete MD with a four-bead protein model (the backbone is represented by three beads corresponding to the amide, alpha-carbon, and the carbonyl groups; the side chain, with the exception of glycin, is represented by only one bead). For the peptides under study, the characteristic oligomer size distributions were obtained, which were in agreement with experimental findings. Aβ_42_ had a high propensity to form pentameric and hexameric structures that could self-associate into higher-order oligomers. Structural analysis revealed that the C-terminal region played a dominant role in Aβ_42_ oligomer formation, whereas Aβ_40_ oligomerization was primarily driven by intermolecular interactions among the central hydrophobic regions. The N-terminal region (2)AEF played a prominent role in Aβ_40_ oligomerization but did not contribute to the oligomerization of Aβ_42_ or the mutants.

Studies conducted in vitro and in vivo suggest that administration of flavonoids, compounds naturally present in many foods including wine and tea, can prevent and reverse Aβ aggregation, but the mechanism of their action is unknown. Lemkul and Bevan [57] employing atomistic, explicit solvent MD simulations investigated the mechanism of Aβ fibril destabilization by morin which is one of the most effective anti-aggregation flavonoids. They used a model of mature Aβ and through the course of 24 simulations found that morin could bind to the ends of the fibrils to block the attachment of an incoming monomeric peptide and can penetrate into the hydrophobic core to disrupt the D23-K28 salt bridges. It also modified the backbone hydrogen bonding.

The stability of Aβ_42_ fibrils and thermodynamics of peptide dissociation were investigated in [58] using all-atom molecular dynamics simulations and pulling one monomer from the pentameric protofibril of Aβ_42_. Results indicated that the presence of water molecules around the D23-K28 salt bridge is crucial to protofibril stability. The extent of packing between hydrophobic residues regulates the level of hydration in the core of the protofibril and thus rigidifies the D23-K28 salt bridge. Such studies explore the mechanism of destabilization of amyloid aggregates which may be important because numerous studies have found that the insoluble fibrillated form of the peptide also contributes to neurotoxicity, although the principal toxic species in Alzheimer’s disease are believed to be the soluble, oligomeric aggregates of Aβ. Membrane disruption and increased ion conductance have been observed in vitro in the presence of Aβ, and it is assumed that the same phenomena occur in the brain neurons of Alzheimer's disease patients. Simulations of Aβ in a membrane bilayer revealed how the peptide interacts with the surrounding lipids and to what extent it affects lipid behavior and contributes to membrane damage. The results showed that Aβ_40_ is capable of disordering the nearby lipids, as well as of decreasing the thickness of the membrane. During simulations the peptide unfolded and finally acquired a disordered, extended conformation allowing for extensive electrostatic and hydrogen bonding interactions with lipids [59]. The stability and conformational dynamics of trimeric and pentameric full-length Aβ_42_ peptides were investigated by Masman et al. [60] for the purpose of defining structural elements influencing their stability. The N-terminal part not detected in NMR was treated as a disordered domain. The models of the oligomer were stable during 100-ns simulations while the β-strand acquired a characteristic twist which facilitated a compact packing of the side chains from the neighboring β-sheets. It seems that the hydrophobic core comprising the β_2_ fragment of the oligomer β-sheet is a stabilizing element in the process of Aβ aggregation. Destabilization of this crucial β-sheet fragment emerges as a prospective target for anti-amyloid drugs.

## Amyloid Can Be Beneficial for Cells and Also Convenient for Engineers

Amyloid fibrils are cross-β-sheet structures that are primarily associated with several neurodegenerative diseases. However, amyloid is also a fundamental nonpathological protein structure (or conformation) utilized by organisms from bacteria to humans. The cross-β-sheet motif is composed of intermolecular β-sheets arranged along the fibril axis with the β strands aligned perpendicularly to the fibril axis. Amyloid fibril formation also provides biologically important entities termed functional amyloids [61] that are present in silkworms [62, 63] and in mammalian skin [64]. It is also known that pituitary hormones are functioning in an amyloid state. Riek and coworkers [65] found that peptide and protein hormones in secretory granules of the endocrine system are stored in an amyloid-like conformation composed of cross-β-sheets. Thus, functional amyloids in the pituitary and other organs can contribute to normal cell and tissue physiology. The hormone amyloids are stored inside the granules, an “inert” membrane container, and the amyloid fibrils dissociate only upon secretion. Additionally, the amyloid aggregation of these hormones must be highly regulated. This regulation may include the processing of prohormones that aggregate more slowly than their hormone counterparts [66] or require the presence of helper molecules to induce aggregation; the latter was demonstrated for prolactin, which lacks a prohormone stage.

Amyloid, a fibrillar quaternary structure, was first discovered in the context of human disease and tissue damage. Therefore it was long thought to be detrimental to the host. However, recent studies have identified functional amyloid fibers in bacteria, fungi, insects, invertebrates, and humans. Nevertheless, physiological amyloidogenesis requires tight regulation to avoid toxicity of the produced amyloids. Diverse physiological applications of amyloids can change our views on the potential treatment of amyloid diseases [61]. The discovery of native amyloids in mammals provides a key insight into the molecular basis of both the physiological and pathological role of amyloids.

The examples of useful amyloids include fungal prions, which are involved in prion replication, the amyloid protein Pmel17 which is involved in biosynthesis of the pigment melanin in mammals, and the factor XII protein of the hemostatic system which is activated by amyloid. The Pmel17 protein forms amyloid fibrils that act as a template and accelerate the covalent polymerization of small reactive molecules into melanin—a critically important biopolymer that protects against a broad range of cytotoxic insults including UV and oxidative damage. The Pmel17 amyloid also appears to play a role in diminishing the toxicity associated with melanin formation by sequestering and minimizing diffusion of highly reactive melanin precursors [64]. The silkmoth chorion protein is also a natural protective amyloid. This is the major component of the eggshell, a structure with extraordinary physiological and mechanical properties. Other natural, protective amyloids are fish chorion, the hydrophobins, and the antifreeze protein from winter flounder.

The phenomenon of the self-assembly of molecules is more and more frequently exploited to invent new supramolecular structures and materials inspired by biological systems such as novel biocompatible polymeric structures with excellent physicochemical properties for new biomedical and industrial applications [62]. A variety of protein and peptide molecules with various amino acid sequences form highly stable and well-organized amyloid assemblies under diverse conditions. They display phase states ranging from liquid crystals to rigid nanotubes. The potential applications of these supramolecular assemblies can be broader than those of synthetic polymers since one can easily introduce biological function in addition to their mechanical properties [67]. Self-assembly is a powerful mechanism for organizing molecular binding blocks into complex structures and aromatic groups can facilitate this process [68, 69]. For example, the Phe-Phe dipeptide motif from Alzheimer’s disease β-amyloid protein was able to self-assemble into peptide-based nanotubes [70]. The Phe-Phe peptide is of special interest due to its ability to form ordered nano-assemblies of unique physical, chemical, and mechanical properties [70, 71]. It was shown that the thermal stability of diphenylalanine peptide nanotubes is significantly higher than that of a nonassembling dipeptide, dialanine. In addition to thermal stability, the peptide nanotubes were chemically stable in many organic solvents. Other aromatic dipeptides can also self-assemble into ordered structures such as tubes, spheres, plates, and hydrogels [71–75]. Moreover, Phe-Phe nanotube-based electrochemical biosensors have shown a large increase in their sensitivity upon the modification of the electrode surfaces with the forest-like nanotube arrays [76–78]. Such bio-inspired materials can be composed of chemically synthesized biomolecules. In the recent work [79], diphenylalanine nanotubes have been used to modify carbon electrodes, by physical vapor deposition of peptide nanotubes, of the electrochemical energy storage devices called supercapacitors. The structural motif of Phe-Phe forms discrete and stiff nanotubes that can be used for production of discrete nanowires with a long persistence length. The same dipeptide building block, made of d-phenylalanine, resulted in the production of enzymatically stable nanotubes [70]. It was shown that a non-charged peptide analogue, Ac-Phe-Phe-NH_2_, self-assembled into similar tubular structures as did diphenylalanine. A similar peptide, diphenylglycine, self-assembled into ordered nanospherical assemblies. Other homo-aromatic dipeptides, in which phenyl side chains were modified with halogen atoms, additional phenyl groups or by alteration of the phenyl groups and naphthyl groups, or by nitro substitutions, were also investigated. In all cases, the well-ordered nanostructures were formed in the shape of tubular, spherical, and two-dimensional structures [71]. Peptide-based nanostructures represent nano-objects of particular interest, as they are biocompatible, can be easily synthesized in large amounts, decorated with functional elements, and used in various biological and non-biological applications.

The significant thermal and chemical stability of the peptide nanotubes could be potentially useful in microelectronics and microelectromechanics as well as for fabrication of functional nanotechnological devices [80]. Amyloids have unusual properties, for instance, rigidities varying over four orders of magnitude depending on the nature of intermolecular forces. The major contribution to their rigidity stems from a generic interbackbone hydrogen bonding network that can be modulated by the variability of side chain interactions [81]. Especially the aromatic residue, side chain interactions play a role in Phe-Phe and related peptide nanotubes and in biological processes such as collagen self-assembly which involves the hydrophobic interactions of Tyr and Phe residues within the C-terminal chain [82, 83]. Usage of bionanostructures in industrial applications requires precise control over self-assembly of monomeric units and the ability to scale up production of these materials. A significant challenge is to control the formation of large, homogeneous arrays of bionanostructures on macroscopic surfaces. The example is the self-assembly of large arrays of aromatic peptide nanotubes using vapor deposition methods. This approach allows controlling of the length and density of the nanotubes by supplying the building blocks from the gas phase. The nanotube arrays can be used to develop high-surface-area electrodes for energy storage applications, microfluidic chips, and also highly hydrophobic self-cleaning surfaces [84]. Other interesting applications are supramolecular gels in nonpolar solvents which are composed of self-assembled nanowires. Such studies highlight the role of self-assembly and gelation in the electronic properties of semiconducting molecular gelators and opens the window for a new class of conducting materials which may find a wide application in organic electronic devices [69]. There was also a proposition of using amyloid fibrils as new nanoscale biomaterials to investigate cell adhesion, migration, and differentiation in vitro. Gras et al. [85] used peptides with an additional segment motif of the biological cell adhesion sequence (RGD) or a control sequence (RAD) at the C-terminus of an 11-residue peptide taken from the amyloidogenic protein transthyretin. The fibrils containing such sequences are bioactive and interact specifically with cells via the incorporated sequences exposed on the fibril surface. Such functionalized fibrils can be systematically altered, so it could be possible to generate nanomaterials based on amyloids to promote interactions for a range of cell types.

One of the most recent and striking examples of the usefulness of a potentially dangerous, fibril-forming protein was described in [86, 87]. The protein is α-synuclein which participates in the Lewy body formation in Parkinson’s disease. The authors fabricated a pea pod-type gold nanoparticle (AuNP) arrangement into one-dimensional chain structures within the dielectric amyloid fibrils of α-synuclein. The assembly units composed of α-synuclein encapsulating AuNPs were manipulated by either hexane or the pH value to induce structural rearrangement within the protein coat. The method of encapsulation of noble metal nanoparticles within dielectric matrices is used to develop fast optoelectric response systems near the surface plasmon resonance frequency. Light energy can be transported through nanoparticles whose sizes are substantially smaller than the wavelength of the corresponding light. These AuNP-embedded amyloid protein nanofibrils exhibited photoconductivity with visible light—such property is crucial for the development of a subwavelength size light-guiding nano-optics systems.

## Conclusions

The genome-wide analysis revealed that self-complementary amyloidogenic segments are found in almost all proteins [6]; however, not all proteins form amyloids. There are 40–50 amyloid-associated diseases identified so far, but only a few proteins were identified to be causative in such diseases. Such an observation may result from the fact that only the most vulnerable proteins convert into amyloids. There are protective mechanisms that shield other proteins from this dangerous behavior. About 500–600 genes/proteins protect young organisms from such diseases, but their role is diminishing with age, so the ultimate goal would be to find a way to restore their protective function. Amyloids can be devastating but also beneficial when kept under control by specific cellular systems. Finally, the unusual properties of amyloid—mechanical, electronic, and other—can be exploited in many industrial applications. These biological nanostructures do not cease to inspire new ideas on how to protect the organism against their detrimental effects but also on how to use them in practical devices.
